# A taxonomic study of *Ooctonus* (Hymenoptera, Mymaridae) from Heilongjiang, China

**DOI:** 10.3897/zookeys.479.9041

**Published:** 2015-01-29

**Authors:** Hai-Feng Bai, Xiang-Xiang Jin, Cheng-De Li

**Affiliations:** 1 School of Forestry, Northeast Forestry University, Harbin, 150040, China

**Keywords:** Chalcidoidea, Mymaridae, *Ooctonus*, taxonomy, new species, China

## Abstract

Five species of *Ooctonus* Haliday (Hymenoptera, Mymaridae) from Heilongjiang Province, China, are reviewed. One species, *Ooctonus huberi* **sp. n.**, is described as new, and four species, *Ooctonus orientalis* Doutt, *Ooctonus saturn* Triapitsyn, *Ooctonus sublaevis* Förster and *Ooctonus vulgatus* Haliday are reported as new to China. A key to the females of the 10 described Chinese species is given. All the specimens are deposited in the insect collections of Northeast Forestry University, China.

## Introduction

*Ooctonus* currently contains 36 described species: one in the Australian region (Perkins 1905), 12 in the Palaearctic region, five in the Oriental region (Triapitsyn 2010), 14 species in the Nearctic region including three also distributed in the Palaearctic region (Huber 2012), three in the Afrotropical region (Huber et al. 2010), and four in the Neotropical region (Huber 2013). Here we describe a new species, record 4 others for the first time from northeast China, and provide a key to females of the 10 *Ooctonus* species known from China.

## Materials and methods

Twenty-three specimens (19 females and 4 males) of *Ooctonus* were collected in Heilongjiang Province, northeast China by sweeping, Malaise traps (MT) or yellow pan traps (YPT). Specimens were dissected and mounted in Canada balsam on slides following the method described by Noyes (1982) and modified for Mymaridae by Huber (1988). Photographs were taken with a digital CCD camera attached to an Olympus BX51 compound microscope, and most measurements were made from slide-mounted specimens using an eye-piece reticle. Total body length excluding ovipositor was measured with an eye-piece reticle from alcohol-preserved specimens before being dissected. All measurements are given in micrometers (μm). Triapitsyn (2010) and Huber (2012) should be consulted for depositories of type specimens, hosts, and literature references to species described from Palaearctic, Oriental, and Nearctic regions. Morphological terminology and abbreviations are those of Huber (2012). All the specimens listed below are deposited in Northeast Forestry University, Harbin, China (NEFU).

### Key to the females of *Ooctonus* species in China

**Table:** 

1	Metacoxa yellowish or brown, different in color from mesosoma	**2**
−	Metacoxa dark brown or black, almost same color as mesosoma	**9**
2	Frenum (Figs 27, 34) smooth medially, reticulate at lateral borders, sometimes also at anterior and posterior margins	**3**
−	Frenum (Figs 5, 13, 19) entirely reticulate, sometimes only faintly so	**4**
3	Funicle with 2 mps on fl_5_ and fl_6_; propodeum (Fig. 27) with median areole well separated from metascutellum by fairly long median carina, but the median carina often incomplete, not extending to anterior margin of propodeum, or almost absent	***Ooctonus sublaevis***
−	Funicle without mps on fl_5_ and fl_6_; propodeum (Fig. 34) with median areole abutting metascutellum; the median carina absent and replaced by the two carinae forming inner margin of dorsolateral areoles	***Ooctonus vulgatus***
4	Mesoscutum (Figs 13, 19) with median longitudinal groove, the groove sometimes very short at posterior margin or extending about 0.7× length of mesoscutum	**5**
−	Mesoscutum (Figs 4, 34) without median longitudinal groove	**6**
5	Funicle with 2 mps on fl_5_ and fl_6_; plica (Fig. 13) bifurcate anteriorly with a long lateral and long medial arm	***Ooctonus orientalis***
−	Funicle without mps on fl_5_ and fl_6_; plica (Fig. 19) bifurcate anteriorly with a short lateral and short medial arm	***Ooctonus saturn***
6	Clava with 8 mps	***Ooctonus insignis* Haliday**
−	Clava with 7 mps	**7**
7	Funicle without mps on fl_6_	***Ooctonus notatus* Walker**
−	Funicle with 1 or 2 mps on fl_6_	**8**
8	Propodeum with median areole separated from metascutellum by median carina; plica with an anterior bifurcation; mesosoma yellow; ovipositor relatively long, at least 1.1× as long as metatibia	***Ooctonus novickyi* Soyka**
−	Propodeum (Fig. 5) with median areole abutting metascutellum; the median carina absent and replaced by the two carinae forming inner margin of dorsolateral areoles; plica without an anterior bifurcation; mesosoma dark brown; ovipositor relatively short, at most 0.9× as long as metatibia	***Ooctonus huberi***
9	Body length about 1 300 μm; mesoscutum without median longitudinal groove or at most with very short one	***Ooctonus himalayus* Subba Rao**
−	Body length about 2 600 μm; mesoscutum with long median longitudinal groove (at least 0.5× length of mesoscutum)	***Ooctonus sinensis* Subba Rao**

## Taxonomy

### Ooctonus huberi

Taxon classificationAnimaliaHymenopteraMymaridae

Bai, Jin & Li sp. n.

http://zoobank.org/9E177EE9-7AD7-4FB2-B8A1-57A71D8B23F4

Figs 1–7 , 8–11

#### Holotype.

♀ (NEFU) Harbin City, Maoershan Town, Mt. Maoershan, 700m. 18.VIII. 2014, Cheng-De Li, Hai-Feng Bai, Xiang-Xiang Jin, YPT.

#### Paratypes.

**3 females, 2 males.** Harbin City, Maoershan Town: Jianlagou. 4–17.VIII. 2014, Cheng-De Li, Hai-Feng Bai, Chao Zhang, Zhi-Guang Wu (2 ♀ ♀, NEFU), MT; same data as holotype (2 ♂ ♂, NEFU); Laoyeling. 16–29.VIII. 2013, Cheng-De Li, Hai-Feng Bai (1 ♀, NEFU), MT.

#### Diagnosis.

Funicle (Fig. 2) with 2 mps at least on fl_4_–fl_8_ and 7 mps on clava; mesoscutum (Fig. 4) without median longitudinal groove; frenum (Figs 4, 5) entirely reticulate; propodeum (Fig. 5) with median areole abutting metascutellum; the median carina absent and replaced by the two carinae forming inner margin of dorsolateral areoles; plica without an anterior bifurcation; petiole 3.54–4.05× as long as wide; ovipositor (Fig. 7) slightly exserted, about 0.9× as long as gaster, and 0.86–0.90× as long as metatibia.

**Figures 1–7.**
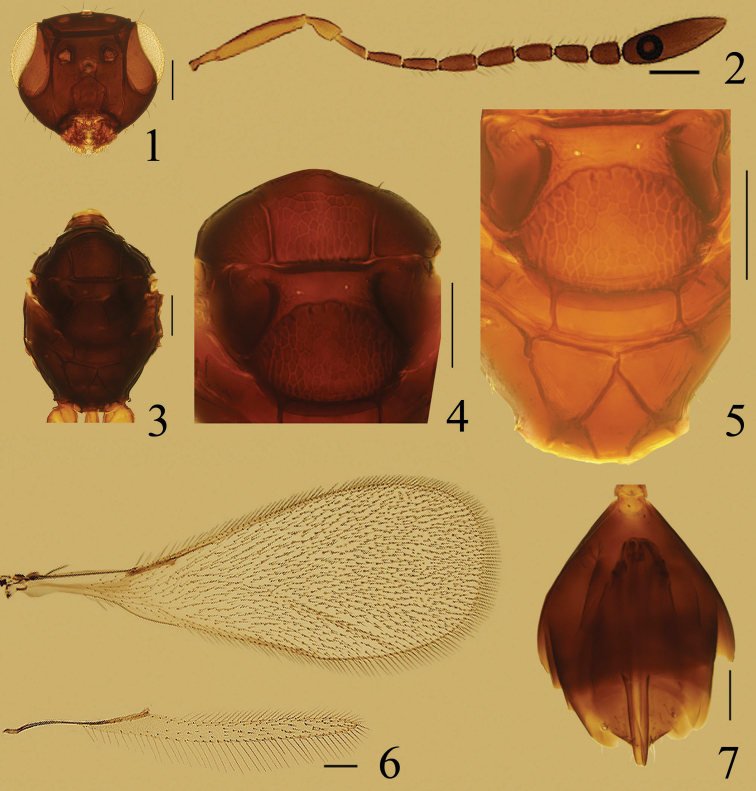
*Ooctonus huberi* sp. n., holotype female (Jianlagou): **1** head, dorsal **2** antenna **3** mesosoma, dorsal **4** mesoscutum and scutellum, dorsal **5** frenum and propodeum, dorsal **6** wings **7** gaster, dorsal. Scale bars = 100 μm.

*Ooctonus huberi* sp. n. runs to *Ooctonus novickyi* in Triapitsyn’s key (2010), and the differences are shown in the key above. The new species is also similar to *Ooctonus lokomotiv*. Both species have 1 mps sometimes on fl_3_ and 2 mps on fl_4_–fl_8_ and have reticulation on mesoscutum and frenum, but *Ooctonus huberi* sp. n. differs from the latter by having 7 mps on the clava (8 mps in *Ooctonus lokomotiv*); petiole 3.54–4.05× as long as wide (2.6–3.3× in *Ooctonus lokomotiv*); and ovipositor 0.86–0.90× as long as metatibia (1.2–1.4× in *Ooctonus lokomotiv*).

Description. Female. Body length 1240–1380. Head and mesosoma dark brown, metasoma brown; scape and pedicel mostly yellow except dorsally dark brown; fl_1_ brown, remainder of funicle dark brown; petiole and legs yellow except apical tarsomere brown.

Head. Head (Fig. 1) width 396–406. Vertex without stemmaticum. Mid ocellus diameter 29–31. Vertex with conspicuous reticulate sculpture; face with faint, inconspicuous reticulate sculpture.

Antenna. Antenna (Fig. 2) with scape 4.61–5.25× as long as wide, slightly longitudinally striate; pedicel slightly longer than fl_1_; funicle with 2 mps on fl_4_–fl_8_ and 7 mps on clava, and sometimes fl_3_ with 1 mps on one antenna. Clava 3.17–3.31× as long as wide, slightly longer than fl_6_–fl_8_ together. Measurements (length/width): radicle 53, scape 199–204/ 38–43, pedicel 65–72/ 36–38, fl_1_ 60–72/ 22–24, fl_2_ 70–79/ 24–26, fl_3_ 72–77/ 26–29, fl_4_ 77–82/ 29–34, fl_5_ 77–79/ 31–36, fl_6_ 72–77/ 31–36, fl_7_ 70–79/ 36–38, fl_8_ 58–65/ 43–46, clava 221–240/ 70–74.

Mesosoma. Mesosoma (Fig. 3) with pronotum weakly sculptured. Mid lobe of mesoscutum (Fig. 4) with meshes raised; scutellar setae long, extending posterior to medially concave frenal line; frenum 0.69–0.75× mesoscutellum length and entirely reticulate. Metanotum with metascutellum smooth. Propodeum (Fig. 5) smooth between carinae and its anterior margin with a stub slightly lateral to lateral margin of metascutellum; median areole abutting metascutellum; the median carina absent and replaced by the two carinae forming inner margin of dorsolateral areoles; plica almost straight, extending almost to anterior margin of propodeum just medial to stub, without an anterior bifurcation but with a slight curved thickening posterior to the stub.

Wings. Fore wing (Fig. 6) length 1415–1512, width 512–585, length/width 2.57–2.76, longest marginal setae 77–84, 0.13–0.15× as long as greatest wing width. Marginal vein length 125–132. Hind wing (Fig. 6) length 1049–1122, width 67–70, length/width 16–17, longest marginal setae 122–125.

Metasoma. Petiole 3.54–4.05× as long as wide, 1.35–1.38× as long as metacoxa, shorter than metacoxa + metatrochantellus. Gaster (Fig. 7) with ovipositor length 455–485, slightly exserted, 0.89–0.91× as long as gaster, and 0.86–0.90× as long as metatibia (515–525).

#### Male.

**Figures 8–11.**
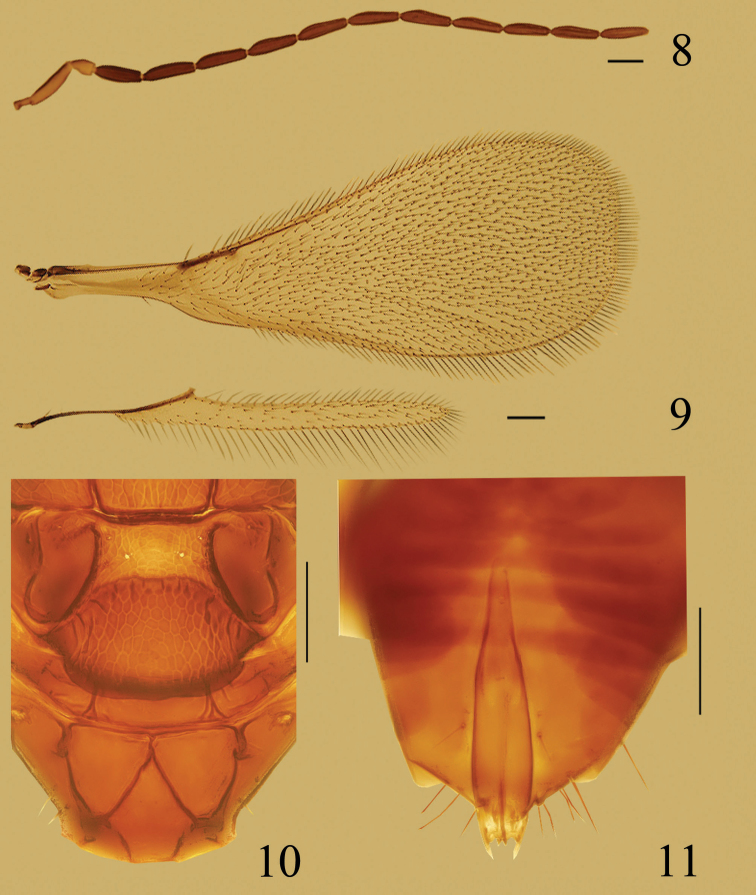
*Ooctonus huberi* sp. n., paratype male (Jianlagou): **8** antenna **9** wings **10** posterior part of mesoscutum to propodeum, dorsal **11** genitalia. Scale bars = 100 μm.

Body length 1230–1310. Mid ocellus diameter 29–31. Antenna (Fig. 8). Measurements, length: radicle 48–50, scape 139–144, pedicel 60–70, fl_1_ 125, fl_2_ 137–144, fl_3_ 142–144, fl_4_ 134–139, fl_5_ 142, fl_6_ 137–142, fl_7_ 137–142, fl_8_ 134–139, fl_9_ 130, fl_10_ 132–137, fl_11_ 134–142. Total flagellar length 1537–1561. Fl_6_ length/width 4.21–4.38, with 7 mps. Fore wing (Fig. 9) length 1463–1512, width 561–585, length/width 2.50–2.70, longest marginal setae 89–101, 0.15–0.18× as long as greatest wing width. Hind wing (Fig. 9) length 1073–1122, width 72, length/width 14.91–15.58, longest marginal setae 132–134, 1.83–1.87× as long as greatest wing width.

#### Host.

Unknown.

#### Etymology.

This species is named in honor of JT Huber, of the Canadian Forest Service, Ottawa, Canada.

### Ooctonus orientalis

Taxon classificationAnimaliaHymenopteraMymaridae

Doutt, 1961

Figs 12–15

Ooctonus orientalis Triapitsyn 2010: 36–40 (redescription, primary type data, distribution).

#### Specimens examined.

3 ♀ ♀. Harbin City, Maoershan Town: Laoyeling. 10–11.VI. 2013, Xiang-Xiang Jin, Si-Zhu Liu, Chao Zhang, sweeping (1 ♀); Laoshan. 12–14.VI. 2013, Xiang-Xiang Jin, Si-Zhu Liu, Chao Zhang, YPT (1 ♀); Jianlagou. 19.VII. 2014, Cheng-De Li, Hai-Feng Bai, Xiang-Xiang Jin, Yan Gao, YPT (1 ♀).

#### Diagnosis.

**Figures 12–15.**
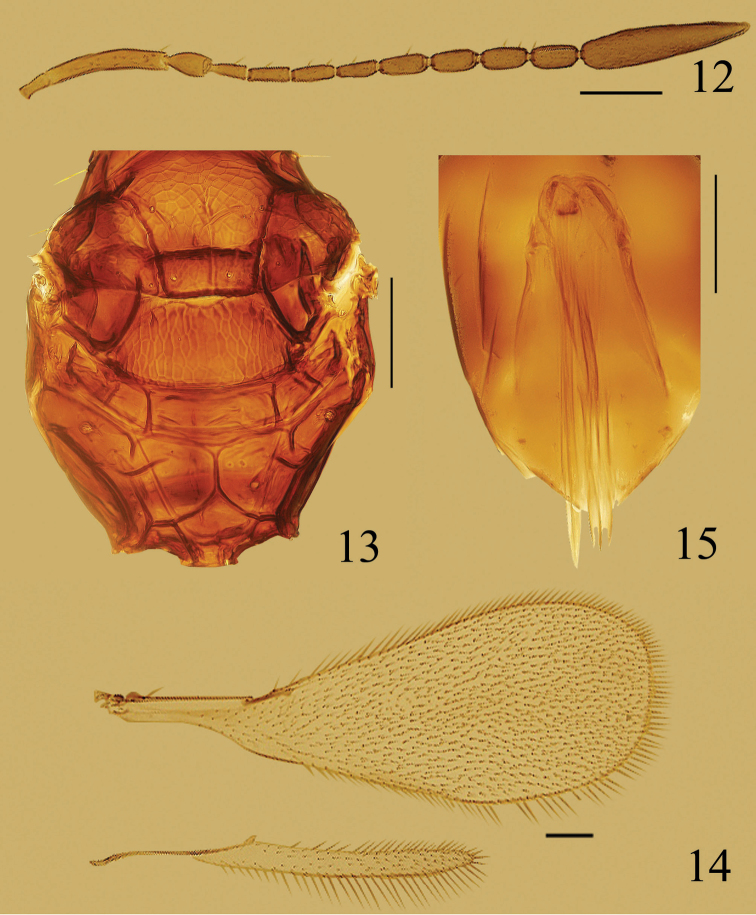
*Ooctonus orientalis*, female (Laoyeling): **12** antenna **13** part of mesosoma, dorsal **14** wings **15** ovipositor. Scale bars = 100 μm.

Funicle (Fig. 12) usually with 2 mps on fl_5_–fl_8_ and 7 mps on clava; mesoscutum (Fig. 13) with median longitudinal groove, the groove sometimes very short at posterior margin of mesoscutum or extending about 0.7× length of mesoscutum; frenum entirely reticulate; propodeum (Fig. 13) with median areole separated from metascutellum by long median carina; plica bifurcate anteriorly with a long lateral and shorter medial arm.

### Ooctonus saturn

Taxon classificationAnimaliaHymenopteraMymaridae

Triapitsyn, 2010

Figs 16–20 , 21–24

Ooctonus saturn Triapitsyn 2010: 36–40 (description, type data, distribution).

#### Specimens examined.

7 ♀ ♀, 2 ♂ ♂. Harbin City, Maoershan Town: Jianlagou. 1–17.VI. 2014, Cheng-De Li, Hai-Feng Bai, Ye Chen, Chao Zhang, MT (3 ♀ ♀); Jianlagou. 4.VIII. 2014, Cheng-De Li, Hai-Feng Bai, Xiang-Xiang Jin, Yan Gao, sweeping (1 ♀); Laoshan. 12–14.VI. 2013, Xiang-Xiang Jin, Si-Zhu Liu, Chao Zhang, YPT (1 ♀); Jianlagou. 17.VI. 2014, Cheng-De Li, Hai-Feng Bai, Ye Chen, Chao Zhang, YPT (2 ♀ ♀, 2 ♂ ♂).

#### Diagnosis.

**Figures 16–20.**
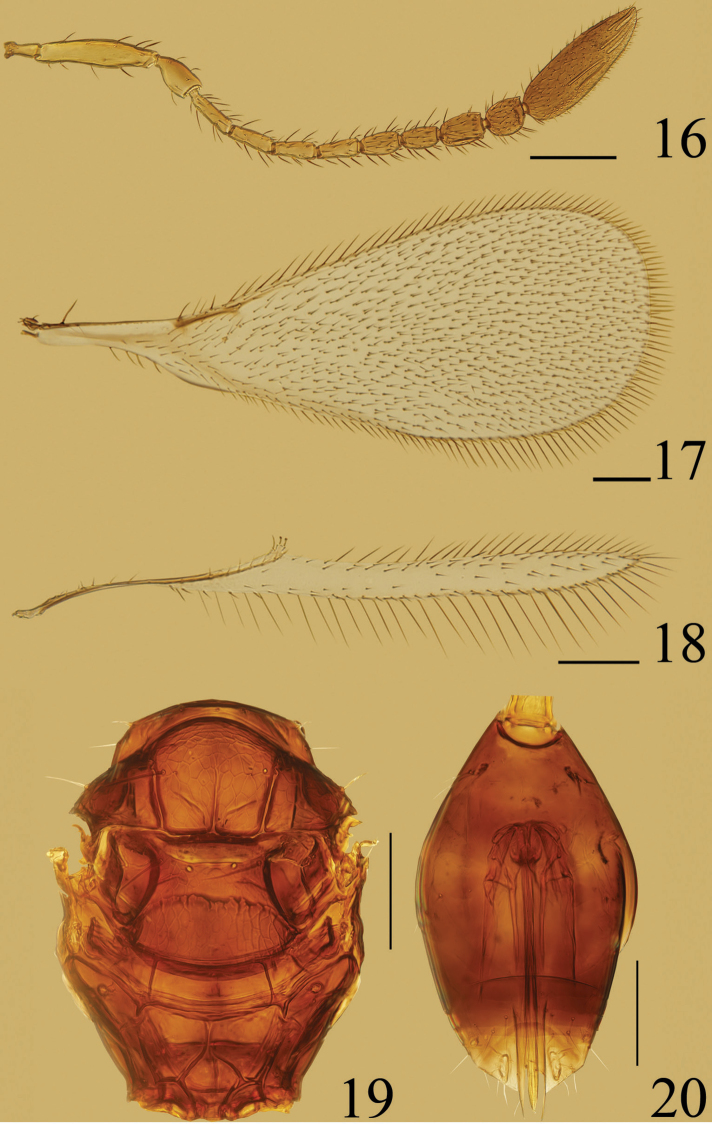
*Ooctonus saturn*, female (Jianlagou): **16** antenna **17** fore wing **18** hind wing **19** mesosoma, dorsal **20** gaster. Scale bars = 100 μm.

Funicle (Fig. 16) with 2 mps on fl_7_ and fl_8_ and 7 mps on clava; mesoscutum (Fig. 19) with median longitudinal groove, the groove sometimes very short at posterior margin of mesoscutum or extending about 0.5× length of mesoscutum; frenum entirely reticulate; propodeum (Fig. 15) with median areole separated from metascutellum by median carina; plica with a short bifurcation anteriorly.

**Figures 21–24.**
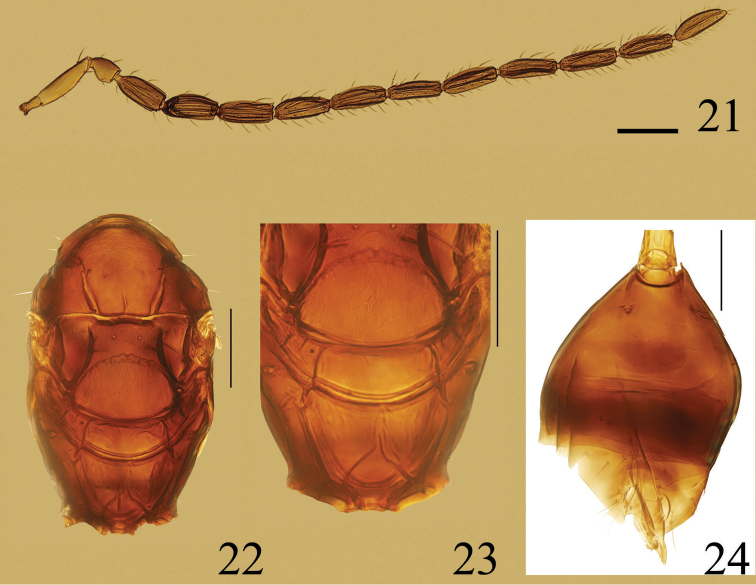
*Ooctonus saturn*, male (Jianlagou): **21** antenna **22** mesosoma, dorsal **23** part of mesosoma, dorsal, **24** gaster. Scale bars = 100 μm.

### Ooctonus sublaevis

Taxon classificationAnimaliaHymenopteraMymaridae

Förster, 1847

Figs 25–29

#### Specimens examined.

4 ♀ ♀: Harbin City, Maoershan Town, Laoyeling. 10–11.VI. 2013, Xiang-Xiang Jin, Si-Zhu Liu, Chao Zhang, sweeping (1 ♀); Yichun City, Wuying Town, Fenglin Natural Reserve. 3–4.VII. 2013, Guo-Hao Zu, Hui Geng, Si-Zhu Liu, Yang Peng, sweeping (3 ♀ ♀).

#### Diagnosis.

**Figures 25–29.**
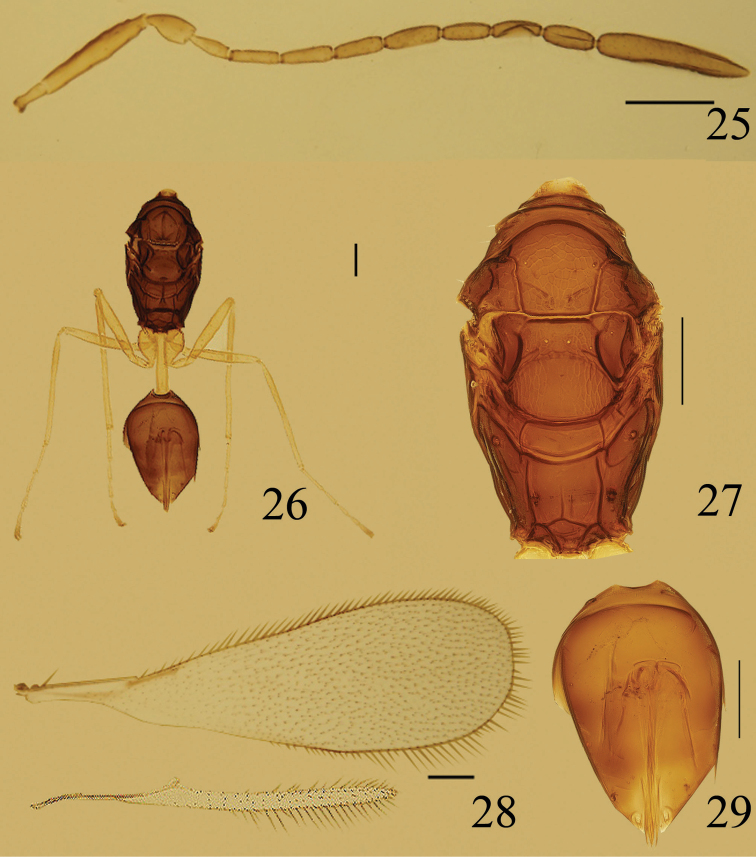
*Ooctonus sublaevis*, female (Fenglin Natural Reserve): **25** antenna **26** body, dorsal **27** mesosoma, dorsal **28** wings **29** gaster. Scale bars = 100 μm.

Funicle (Fig. 25) usually with 2 mps on fl_5_–fl_8_ (occasionally fl_6_ with just 1 mps) and 7 mps on clava; mesoscutum (Fig. 27) usually without median longitudinal groove, rarely with a very short groove; frenum with weak reticulate sculpture; propodeum (Fig. 27) with median areole well separated from metascutellum by fairly long median carina, but the median carina often incomplete, not extending to anterior margin of propodeum, or almost absent; plica straight or slightly curved outward and not divided anterodorsally.

### Ooctonus vulgatus

Taxon classificationAnimaliaHymenopteraMymaridae

Haliday, 1833

Figs 30–35

#### Specimen examined.

1 ♀. Harbin City, Maoershan Town, Laoyeling. 17.VI. 2014, Cheng-De Li, Hai-Feng Bai, Guo-Hao Zu, Ye Chen, sweeping.

#### Diagnosis.

**Figures 30–35.**
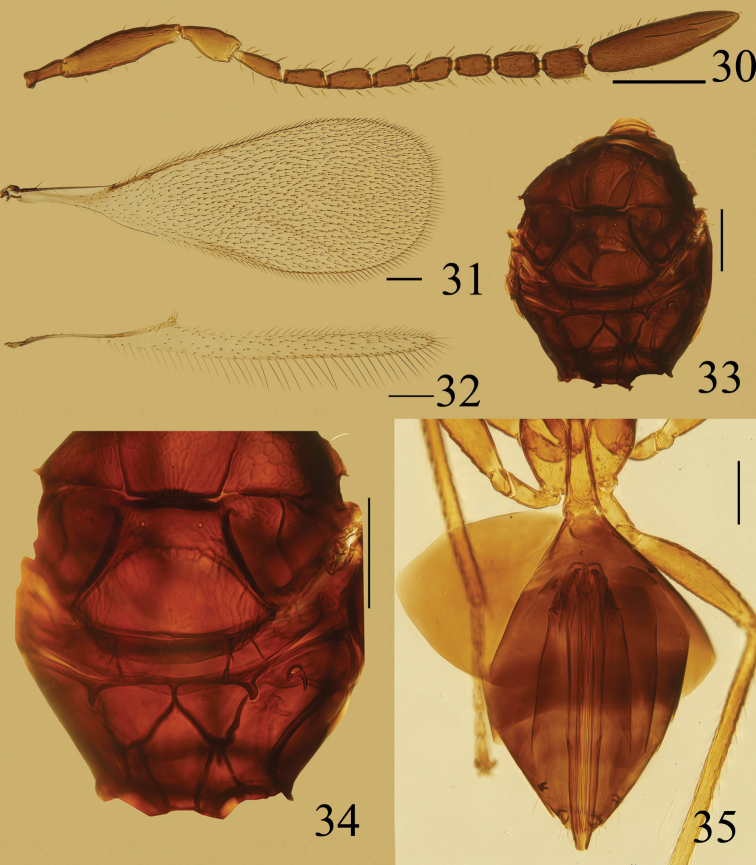
*Ooctonus vulgatus*, female (Laoyeling): **30** antenna **31** fore wing **32** hind wing **33** mesosoma, dorsal **34** posterior part of mesoscutum to propodeum, dorsal **35** gaster. Scale bars = 100 μm.

Funicle (Fig. 30) with 2 mps on fl_7_ and fl_8_ and 7 mps on clava; mesoscutum (Fig. 33) without median longitudinal groove; frenum mostly smooth, except for obscure sculpture at lateral borders and sometimes also at anterior margin; propodeum (Fig. 34) with median areole abutting metascutellum; the median carina absent and replaced by the two carinae forming inner margin of dorsolateral areoles; plica almost straight and not divided anterodorsally, ending just anterior and medial to stub.

## Supplementary Material

XML Treatment for Ooctonus huberi

XML Treatment for Ooctonus orientalis

XML Treatment for Ooctonus saturn

XML Treatment for Ooctonus sublaevis

XML Treatment for Ooctonus vulgatus
